# Randomized Controlled Trial of Group‐Blended and Individual‐Unguided Online Mindfulness‐Based Cognitive Therapy to Reduce Psychological Distress in People With Cancer

**DOI:** 10.1002/pon.70286

**Published:** 2025-09-19

**Authors:** Nasim Badaghi, Linda Kwakkenbos, Judith Prins, Rogier Donders, Saskia Kelders, Anne Speckens

**Affiliations:** ^1^ Department of Psychiatry Radboud University Medical Center Nijmegen the Netherlands; ^2^ Department of IQ Health Radboud University Medical Center Nijmegen the Netherlands; ^3^ Department of Clinical Psychology Behavioural Science Institute Radboud University Nijmegen the Netherlands; ^4^ Department of Medical Psychology Radboud University Medical Center Nijmegen the Netherlands; ^5^ Department of Psychology, Health, and Technology University of Twente Enschede the Netherlands; ^6^ Optentia Research Unit North West University Vanderbijlpark South Africa

**Keywords:** cancer, mindfulness, oncology, psycho‐oncology, randomized controlled trial

## Abstract

**Objective:**

Online mindfulness‐based cognitive therapy (eMBCT) can reduce psychological distress in people with cancer, but adherence and scalability could be improved. Through co‐creation, we developed two eMBCT formats: group‐blended and individual‐unguided. This trial compared the effects of the two eMBCT to care as usual (CAU) on psychological distress and other mental health outcomes in people with cancer.

**Methods:**

In this parallel, three‐armed randomized controlled trial, people with cancer were randomly allocated to group‐blended eMBCT, individual‐unguided eMBCT, or CAU. Participants completed baseline, mid‐treatment, post‐treatment, and 3 months follow‐up assessments. The primary outcome analyzed in the intention‐to‐treat (ITT) population was psychological distress (Hospital Anxiety and Depression Scale) at post‐treatment.

**Results:**

In total, 186 participants were randomized to group‐blended eMBCT (*N* = 57), individual‐unguided eMBCT (*N* = 75), or CAU (*N* = 54). Most participants were female (81%) with breast cancer (49%), and treated with curative intent (76%). In ITT analyses, group‐blended eMBCT participants reported significantly lower levels of psychological distress at post‐treatment (Cohen's *d* = 0.38) and follow‐up (Cohen's *d* = 0.64) than those receiving CAU, while individual‐unguided eMBCT participants only had significantly less psychological distress at follow‐up (Cohen's *d* = 0.48). Additionally, participants in group‐blended eMBCT had less rumination and greater mindfulness, decentering, and self‐compassion than those in CAU at follow‐up. Participants in individual‐unguided eMBCT had greater decentering than those in CAU at post‐treatment and follow‐up, and less rumination and greater mindfulness skills than CAU at follow‐up.

**Conclusions:**

Compared to CAU, both eMBCT conditions were effective in reducing psychological distress and could be an accessible, and potentially low‐cost intervention to reduce distress in people with cancer.

**Trial registration:**

Dutch Registry CCMO, NL73117.091.20; clinicaltrials.gov, NCT05336916

## 1 Background

The number of cancer diagnoses is increasing, and so are survival rates [1]. Globally, approximately one in five people will develop cancer during their lifetime [1]. There is an increasing number of people living with or beyond cancer, often facing its lasting consequences, including psychological challenges. It has been reported that up to 38% of them experience substantial psychological distress [2].

Different psychological interventions exist to support people with cancer to cope with the disease [3]. The American Society of Clinical Oncology (ASCO) recently issued guidelines recommending mindfulness‐based interventions (MBIs) as the first‐line treatment, supported by the highest level of evidence [4]. Mindfulness is commonly defined as present‐moment, non‐judgmental awareness of one's experiences [5]. Mindfulness is a central in MBIs, which aim to cultivate this awareness through a combination of practices such as the body scan, awareness of breathing, mindful movement (e.g., yoga), (group) reflections, and psychoeducation. Although MBIs have consistently demonstrated to be effective in people with cancer [6], their adherence and scalability could be improved [7].

In a previous trial from our team, an individual, therapist‐assisted online MBCT (eMBCT) was more effective than care as usual (CAU) in reducing psychological distress, fear of cancer recurrence, and rumination in distressed people with cancer. Therapist‐assisted eMBCT also improved mindfulness skills, mental health‐related quality of life, and well‐being [7]. However, drop‐out rates in eMBCT were higher than in face‐to‐face MBCT (30% vs. 17%, respectively) and participants reported asynchronicity in communication and lack of peer support as barriers to eMBCT participation [7]. Additionally, mindfulness teachers reported a high time investment for providing individual online guidance [8].

To enhance effectiveness, adherence, and scalability of eMBCT, we developed two online formats—group‐blended and individual‐unguided—through co‐creation with people with cancer, mindfulness teachers, researchers, and eHealth experts [9, 10]. The group‐blended eMBCT included four video‐conferencing sessions led by mindfulness teachers, fostering peer support, interaction, and flexibility. The individual‐unguided eMBCT was self‐guided, featuring an automated avatar, enabling greater flexibility and cost‐effective scalability [9, 10]. A non‐randomized pilot study confirmed both formats were feasible and acceptable to participants [10]. Limited healthcare resources, financial, infrastructural an workforce‐related, often constrain the ability to reach all those in need. This highlights the urgency of developing solutions that can effectively reach broader populations.

The aim of this three‐arm randomized controlled trial (RCT) was to evaluate the effect of group‐blended and individual‐unguided eMBCT, compared to CAU, in people with cancer on psychological distress and other mental health outcomes both post‐treatment and at 3 months follow‐up. In addition, we aimed to explore potential mediators of treatment effects.

## 2 Methods

### 2.1 Trial Design

The Buddy trial was a three‐arm, parallel RCT comparing group‐blended and individual‐unguided eMBCT with CAU in people with cancer and cancer survivors. This study was approved by the ethical review board of Radboud University Medical Center (CMO Arnhem‐Nijmegen, number: NL73117.091.20), registered in the Dutch Registry CCMO (NL73117.091.20) prior starting recruitment, and on ClinicalTrials.gov (NCT05336916) prior to analyses. All participants provided written informed consent [9]. We published the trial protocol before trial completion [9] and followed CONSORT, CONSORT‐EHEALTH, and TiDieR guidelines for reporting [11, 12].

### 2.2 Participants

Eligible participants were adults who had been diagnosed with cancer (any type, stage, or time since diagnosis), had internet access, computer skills, and proficiency in Dutch. Exclusion criteria were (1) prior participation in an MBI (> four sessions); (2) severe psychiatric comorbidities requiring acute treatment (e.g., psychosis, suicidality); (3) drugs or alcohol dependence; (4) severe cognitive impairments; or (5) unwillingness to be randomized to one of the three trial conditions.

To maximize inclusivity and reflect the target population for real‐world implementation in the Dutch healthcare system post‐trial, we did not screen for psychological distress, in line with the preferences of people with cancer involved in the pilot study.

### 2.3 Procedure

The study was conducted at the Radboudumc Expertise Center for Mindfulness, Nijmegen, The Netherlands. We recruited participants between January 2021 and September 2023 through posters and flyers in healthcare settings, regional newspaper ads, online posts on cancer‐related websites, and social media. Interested individuals contacted the research team through the Buddy trial website. A team member provided study details, screened for eligibility, and collected demographic and clinical data via phone call. Eligible participants received written study information and an informed consent form via mail and email. Those who returned the signed consent form were enrolled, completed baseline assessments via Castor EDC (Castor EDC is a secure, cloud‐based electronic data capture platform designed for managing clinical trial data and research workflows), and were then randomized, stratified for cancer type (breast cancer vs. other) and treatment intent (curative vs. palliative). To encourage participant retention and outcome measure completion, we sent reminders through email and WhatsApp.

### 2.4 Intervention and Comparator

Group‐blended and individual‐unguided eMBCT were based on the MBCT program by Segal et al. [13]. Both conditions followed the prototypical 8‐week MBCT (2,5‐h sessions per week), with formal and informal mindfulness practices, reflections, homework (30 min to an hour daily), and a silent day. In a co‐creation process, we adapted the program by including psychoeducation about cancer and grief and modifying the movement exercises for people with cancer. Intervention development and session content have been published previously [9, 10].

The eMBCT programs were hosted on Minddistrict. After creating an account, participants accessed Minddistrict via a web browser or app. Session content across both intervention conditions was identical, but the delivery differed. In group‐blended eMBCT, sessions one, three, five, and eight were live group sessions with a mindfulness teacher via videoconferencing, while the others were platform‐based. In individual‐unguided eMBCT, the sessions were all platform‐based and facilitated by an automated avatar. Participants in both conditions could join with a significant other.

The group‐blended sessions were led by healthcare professionals experienced in psycho‐oncology, meeting the qualification criteria of the Dutch‐Flemish Association of Mindfulness Teachers, aligned with UK standards [14]. They attended regular peer discussions supervised by a senior mindfulness teacher (AS).

CAU participants completed trial outcome questionnaires but did not have access to the eMBCT program. Participants in all conditions could have any form of medical, psychological, or paramedical care during the study period, except for MBIs outside of the study context.

### 2.5 Randomization and Blinding

A unique identification code for each participant and stratification variables were entered in Castor EDC, which immediately randomized them with complete allocation concealment using permuted blocks of random sizes (6, 12, 18). Expecting higher dropout in the individual‐unguided condition, participants were initially randomized 1:2:1 to group‐blended eMBCT, individual‐unguided eMBCT, or CAU. After a year, the ratio was adjusted to 1:1:1 due to similar dropout rates [10]. Participants were not blinded to intervention status, which is unavoidable in psychological interventions trials [15]. Data analysis was performed by an investigator with no contact with participants and no involvement in randomization.

### 2.6 Outcome Measures

Participants completed outcome measures at baseline (T0), post‐treatment (T1), and 3 months follow‐up (T2). The primary outcome was psychological distress at post‐treatment, assessed using the Hospital Anxiety and Depression Scale (HADS) [16]. The HADS is a self‐report scale developed to assess anxiety and depression in medical outpatients.

Secondary outcomes included the Fear of Cancer Recurrence Inventory (FCRI) [17]; the fatigue severity subscale of the Checklist Individual Strength (CIS) [18]; the rumination subscale of the Rumination and Reflection Questionnaire (RRQ) [19]; the Five Facet Mindfulness Questionnaire‐Short Form (FFMQ‐SF) [20]; the decentering subscale of the Experiences Questionnaire (EQ) [21]; the Self‐Compassion Scale‐Short Form (SCS‐SF) [22]; and the Mental Health Continuum‐Short Form (MHC‐SF) [23]. The RRQ, FFMQ‐SF, EQ, and SCS‐SF were also completed at mid‐treatment (between sessions four and five) as potential mediators of treatment effects. Detailed information about each questionnaire is available in the trial protocol [9].

Engagement with the intervention was measured with the Twente Engagement with E‐health Technologies Scale (TWEETS) [24]. The TWEETS was developed to measure engagement with digital health tools. It was completed by eMBCT participants in both conditions at mid‐treatment and T1. eMBCT usage was automatically logged in the Minddistrict platform, and calculated as the average completion percentage per session in each eMBCT group. For group‐blended participants, mindfulness teachers also recorded presence for each videoconferencing session.

Mental health care utilization was measured using the Trimbos/iMTA questionnaire for Costs associated with Psychiatric illness (TiC‐P) [25]. Only the questions regarding the number of visits in the past 3 months to mental healthcare professionals (.e., mental health care institution, practice nurse, social worker, psychologist, psychiatrist, addiction consultation center, self‐help group) were used.

### 2.7 Sample Size

Sample size was calculated based on a three‐group ANCOVA, with psychological distress (HADS) at post‐treatment as the primary outcome. We used a closed testing procedure for pairwise comparisons between group‐blended eMBCT versus CAU, and individual‐unguided eMBCT versus CAU [26]. Previously, we found that the effect size (ES) of face‐to‐face MBCT was *d* = 0.45 and that of eMBCT was *d* = 0.71 [7]. We expected the ES of group‐blended eMBCT to be the mean of these two, that is *d* = 0.58 and that of the individual‐unguided eMBCT versus CAU the smaller of these two, that is *d* = 0.45. Alpha was set at 0.05 and the Pearson correlation between baseline and post‐treatment outcomes was assumed to be 0.50 [7]. For a power of 0.80, a minimal group size of 65 was required for CAU. This resulted in a power of 0.95 of the three‐group ANCOVA, with a power of 0.92 for the comparison individual‐unguided eMBCT and CAU, and 0.82 for the comparison group‐blended eMBCT and CAU. In accordance with our prior trial, we anticipated 20% dropout in group‐blended eMBCT, 40% in individual‐unguided eMBCT, and 0% in CAU [7]. Thus, we targeted to include 254 participants: 81 in group‐blended eMBCT, 108 in individual‐unguided eMBCT, and 65 in CAU.

### 2.8 Statistical Analysis

#### 2.8.1 Effect of Intervention

We conducted descriptive analyses of participants' baseline demographic and clinical characteristics. Average completion rates of the sessions between group‐blended and individual‐unguided eMBCT were compared using independent samples *t*‐tests. Mental health care utilization between trial conditions was compared using Pearson X^2^ test. To examine engagement changes over time by condition, we conducted a mixed ANOVA with time (mid‐vs. post‐treatment) as the within‐subject factor and condition (group‐blended vs. individual‐unguided) as the between‐subject factor, testing main effects and their interaction.

The primary outcome analysis compared HADS total scores at post‐treatment (T1) between group‐blended eMBCT versus CAU and individual‐unguided eMBCT versus CAU in the intention‐to‐treat (ITT) sample. We used ANCOVA with a closed testing procedure, including trial arm as a between‐subject factor and baseline HADS scores and stratification variables as covariates.

Secondary outcomes at T1 and primary and secondary outcomes at 3 months follow‐up (T2) were analyzed similarly. We calculated Cohen's *d* effect sized (ES) [27], interpreting them as small (0.2–0.5), medium (0.5–0.8), or large (> 0.8) [27]. All analyses were two‐sided with an alpha of 0.05. To minimize the possibility of bias from missing outcome data, we used multiple imputation by chained equations (20 imputed datasets, 15 cycles each) using the mice package [28]. Per‐protocol analyses (participants completing ≥ four eMBCT sessions) were conducted as sensitivity analyses. Statistical analyses were performed in SPSS version 29 by NB and reviewed by RD, LK, and AS. The statistical analysis plan (SAP) is available in the online supplementary material.

### 2.9 Mediation Analysis

We conducted mediation analyses on the per‐protocol sample following Hayes and Rockwood [26]. For each mediator (RRQ, FFMQ, EQ, and SCS‐SF), we conducted mediation analyses including the baseline values of both the mediator and the outcome (HADS) as covariates [26]. HADS at post‐treatment was the dependent variable, with baseline HADS entered as a covariate. Exploratory analyses used HADS at 3‐month follow‐up as the outcome, with baseline HADS and baseline mediator scores included as covariates. Separate mediation models were conducted comparing group‐blended eMBCT versus CAU and individual‐unguided eMBCT versus CAU, using 5000 bootstrap samples to generate 95% confidence intervals.

Mediators were assessed at sessions four to five to ensure temporal precedence, balancing the risk of measuring too early, before relevant processes emerge, and too late, when outcome changes could confound mediation [29].

#### 2.9.1 Protocol Amendments

Protocol amendments are described in the online supplementary material.

## 3 Results

### 3.1 Participants

**FIGURE 1:**
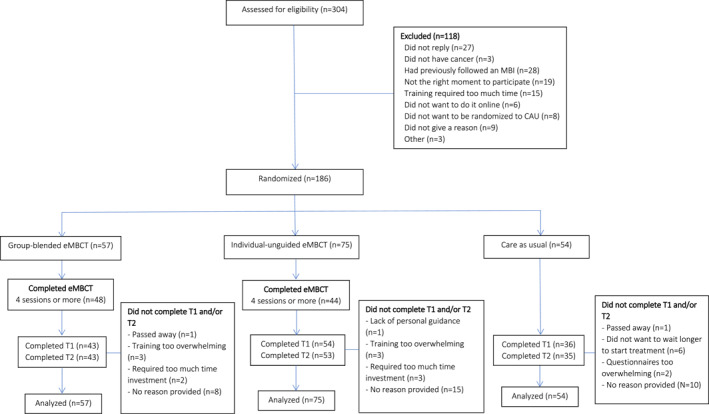
Participant flow chart of the Buddy three‐armed RCT.

Between January 2021 and September 2023, 186 participants were enrolled (see Figure 1). Enrollment was stopped before reaching the targeted sample size of 254 participants due to slower‐than‐expected recruitment post‐COVID‐19 and project grant constraints [9].

Of the 186 participants, 57 (31%) were allocated to group‐blended eMBCT, 75 (40%) to individual‐unguided eMBCT, and 54 (29%) to CAU. Baseline sociodemographic and clinical characteristics were similar across the three groups (see Table 1). Most participants were female (81%) with a mean age of 52.6 (SD = 11.4) years who had completed a high level of education (61%). Almost half of the participants had breast cancer and approximately three quarters received or were receiving treatment with curative intent. At baseline, elevated HADS scores (≥ 11) were observed in 45 of 57 participants (78.9%) in the group‐blended condition, 53 of 75 (70.7%) in the individual‐unguided condition, and 47 of 54 (87.0%) in the CAU condition. Significant others of 16 participants (28%) in the group‐blended condition and 28 participants (37%) in the individual‐unguided condition joined the program.

**TABLE 1:** Participants' baseline sociodemographic and clinical characteristics (*N* = 186).

Characteristic	All (*N* = 186)	Group‐blended (*N* = 57)	Individual‐unguided (*N* = 75)	Care as usual (*N* = 54)
Sex, *N* (%)				
Female	150 (80.6)	45 (78.9)	61 (81.3)	44 (81.5)
Male	36 (19.4)	12 (21.1)	14 (18.7)	10 (18.5)
Age in years, mean (SD)	52.6 (11.4)	54.6 (10.5)	51.2 (11.8)	52.5 (11.8)
Diagnosis, *N* (%)				
Breast cancer	91 (48.9)	27 (47.4)	38 (50.7)	26 (48.1)
Blood cancer	12 (6.5)	5 (8.8)	3 (4.0)	4 (7.4)
Skin cancer	11 (5.9)	5 (8.8)	3 (4.0)	3 (5.6)
Intestine cancer	10 (5.3)	3 (5.3)	4 (5.3)	3 (5.6)
Ovarian	8 (4.3)	2 (3.5)	3 (4.0)	3 (5.6)
Prostate cancer	8 (4.3)	4 (7.0)	4 (5.3)	0 (0.0)
Colon cancer	5 (2.7)	1 (1.8)	2 (2.7)	2 (3.7)
Other	41 (22.1)	10 (17.4)	18 (24.0)	13 (24.1)
Treatment intent, *N* (%)				
Curative	142 (76.3)	44 (77.2)	56 (74.7)	42 (77.8)
Palliative	44 (23.7)	13 (22.8)	19 (25.3)	12 (22.2)
Occupation, *N* (%)				
Employed	117 (62.8)	36 (63.1)	48 (64.1)	33 (61.1)
Retired	28 (15.1)	8 (14.0)	12 (16.0)	8 (14.8)
On disability	16 (8.6)	5 (8.8)	9 (12.0)	2 (3.7)
Houseman/‐wife	12 (6.5)	3 (5.3)	4 (5.3)	5 (9.3)
Unemployed	5 (2.7)	2 (3.5)	1 (1.3)	2 (3.7)
Other (not specified)	8 (4.3)	3 (5.3)	1 (1.3)	4 (7.4)
Level of education, *N* (%)				
High	114 (61.3)	35 (61.4)	46 (61.3)	33 (61.1)
Middle	64 (34.4)	21 (36.9)	26 (34.7)	17 (31.5)
Low	4 (2.2)	0 (0.0)	3 (4.0)	1 (1.9)
Other (not specified)	4 (2.2)	1 (1.8)	0 (0.0)	3 (5.6)
Time since diagnosis in years, *N* (%)				
1–1	13 (7.0)	5 (8.8)	3 (4.0)	5 (9.3)
1–3	78 (41.9)	23 (40.4)	33 (44.0)	22 (40.7)
3–5	42 (22.6)	14 (24.6)	15 (20.0)	13 (24.1)
> 5	53 (28.5)	15 (26.3)	24 (32.0)	14 (25.9)
Married/in a relationship, *N* (%)				
Yes	121 (65.1)	34 (59.6)	54 (72)	33 (61.1)
No	65 (34.9)	23 (40.4)	21 (28)	21 (38.9)

### 3.2 Mental Health Care Utilization

As shown in Table 2, *N* = 126 participants used some form of mental healthcare between baseline and T1, with a mean number of 2.8 (SD = 4.4) visits. There were no significant differences in health care utilization between the trial conditions.

**TABLE 2:** Mental health care utilization between baseline and post‐treatment (T1) for group‐blended eMBCT, individual‐unguided eMBCT, and CAU groups.

Utilization	All (*N* ^−^ = 126)		Group‐blended (*N* = 42)		Individual‐unguided (*N* = 51)		CAU (*N* = 33)		*p* ^b^
	Participants *N* (%)	Visits mean (SD)	Participants *N* (%)	Visits mean (SD)	Participants *N* (%)	Visits mean (SD)	Participants *N* (%)	Visits mean (SD)	
Practice nurse	27 (21)	0.57 (1.59)	10 (24)	0.60 (1.49)	7 (14)	0.37 (1.13)	10 (30)	0.85 (2.22)	0.66
Social worker	15 (12)	0.30 (1.25)	9 (21)	0.67 (1.99)	2 (4)	0.10 (1.13)	4 (12)	0.15 (0.44)	0.15
Self‐help	19 (15)	0.66 (1.85)	7 (17)	0.90 (2.44)	6 (12)	0.35 (1.04)	6 (18)	0.82 (1.94)	0.79
Secondary care^a^	49 (39)	1.24 (2.25)	15 (36)	1.29 (2.43)	21 (41)	1.29 (2.25)	13 (39)	1.09 (2.05)	0.56

*Note:* *N* ^−^ = 126 due to missing data.

Abbreviation: SD, standard deviation.

^a^ Psychologist own practice, psychologist or psychiatrist in the hospital, regional institution for outpatient mental health care, consultation office for alcohol and drugs.

^b^ Pearson x2 test comparing the number of participants between groups.

### 3.3 Adherence and Engagement With Intervention

**FIGURE 2:**
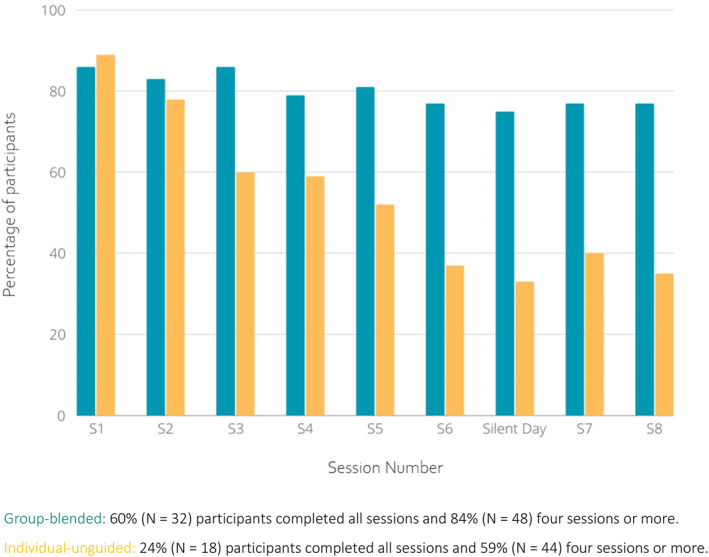
Percentage of participants completing each session by group.

Group‐blended eMBCT participants completed on average more sessions (7.5, SD = 2.7) than those in individual‐unguided eMBCT (5.0, SD = 3.3; *p* < 0.001). The percentage of participants completing four session or more was also higher in the group‐blended (*N* = 48, 84%) than in the individual‐unguided condition (*N* = 44, 59%). Attendance rates were stable in the group‐blended eMBCT but declined over time in the individual‐unguided eMBCT (See Figure 2).

Among participants who completed the TWEETS, a total score of 22.5 or higher can be interpreted as indicating high engagement, based on an item‐level threshold of 2.5 across the nine‐item scale [30]. Against this benchmark, both conditions fell within the medium to high engagement range at both time points.

The mixed‐design ANOVA showed no main effects of time or condition, but a significant time × condition interaction, F (1, 87) = 7.24, p = 0.009. Paired *t*‐tests revealed increased engagement in the group‐blended condition from mid‐ (*M* = 23.4) to post‐treatment (*M* = 25.0), p = 0.030, and a non‐significant decrease in the individual‐unguided condition. See Supporting Information S1 for full statistics.

### 3.4 Trial Outcomes

Outcome data were obtained for 43 (75%) participants in the group‐blended condition at T1 and T2 (see Figure 1). In the individual‐unguided condition, 54 (72%) participants completed T1 and 53 (70%) T2. In the CAU group, 36 (67%) participants completed T1 and 35 (65%) T2. Participants who did not complete post‐treatment questionnaires more often had a type of cancer other than breast cancer (60% vs. 47%, respectively) and more often had received palliative treatment than those completing them (30% vs. 19%, respectively). They also tended to have higher baseline mean HADS scores (17.0 (SD = 6.3) versus 15.2 (SD = 6.8), *p* = 0.08).

The primary ITT ANCOVA showed a significant group effect on post‐treatment HADS scores (*F*‐values for imputed data sets ranging from 1.19 to 6.55), with partial eta squared values ranging from 0.013 to 0.068, and *p* < 0.001 for all imputed datasets. Post‐hoc pairwise comparisons showed that participants in the group‐blended eMBCT had significantly lower HADS scores than those receiving CAU at post‐treatment. The mean difference between individual‐unguided eMBCT and CAU was non‐significant. See Table 3.

**TABLE 3:** Primary and secondary outcome measures at baseline, post‐intervention, and 3‐month follow‐up (intention to treat).

Group							Pairwise comparisons			
Outcome measure	Group‐blended eMBCT (*N* = 57)		Individual‐unguided eMBCT (*N* = 75)		CAU (*N* = 54)		Group‐blended eMBCT versus. CAU		Individual‐unguided eMBCT versus. CAU	
	M	SE	M	SE	M	SE	Mean difference (95% CI)	Cohen's *d* (95% CI)	Mean difference (95% CI)	Cohen's *d* (95% CI)
Psychological distress										
T0	15.35	0.19	15.51	0.17	16.31	0.19				
T1 imputed	11.11	0.76	12.37	0.73	13.87	0.88	**−2.75 (−5.08; −0.43)**	**0.38 (0.01; 0.76)**	−1.50 (−3.82; 0.82)	0.28 (−0.13; 0.69)
T2 imputed	10.25	0.71	10.97	0.59	13.34	0.73	**−3.08 (−5.13; −1.04)**	**0.64 (0.18; 1.06)**	**−2.37 (−4.24; −0.49)**	**0.48 (0.09; 0.87)**
Fear of cancer recurrence										
T0	80.14	0.64	77.58	0.63	77.28	0.67				
T1 imputed	69.84	2.10	72.67	1.93	71.94	2.34	−2.10 (−8.36; 4.16)	0.01 (−0.40; 0.42)	0.72 (−4.79; 6.24)	−0.04 (−0.41; 0.32)
T2 imputed	66.62	2.33	71.61	2.08	70.68	2.50	−4.06 (−10.52; 2.41)	0.11 (−0.29; 0.52)	0.93 (−5.14; 6.99)	−0.05 (−0.42; 0.32)
Fatigue severity										
T0	38.05	0.34	35.72	0.29	38.31	0.31				
T1 imputed	32.01	1.43	35.51	1.27	35.51	1.50	−3.05 (−7.56; 0.55)	0.32 (−0.10; 0.75)	0.00 (−3.77; 3.78)	0.14 (−0.25; 0.53)
T2 imputed	31.33	1.40	33.53	1.26	33.90	1.47	−2.57 (−6.45; 1.31)	0.24 (−0.17; 0.65)	−0.55 (−4.33; 3.23)	0.19 (−0.21; 0.58)
Rumination										
T0	3.40	0.02	3.28	0.02	3.43	0.02				
T1 imputed	3.01	0.08	2.99	0.07	3.15	0.10	−0.14 (−0.39; 0.12)	0.22 (−0.23; 0.68)	−0.16 (−0.40; 0.09)	0.36 (−0.07; 0.79)
T2 imputed	3.00	0.09	3.01	0.08	3.26	0.09	**−0.26 (−0.51; −0.01)**	0.38 (−0.04; 0.80)	**−0.26 (−0.49; −0.02)**	**0.48 (0.08; 0.87)**
Mindfulness skills										
T0	79.53	0.31	78.15	0.28	77.77	0.36				
T1 imputed	83.55	1.29	84.94	1.11	82.84	1.54	0.71 (−3.35; 4.76)	0.16 (−0.27; 0.59)	2.09 (−1.59; 5.78)	0.21 (−0.18; 0.60)
T2 imputed	86.19	1.24	84.37	1.09	80.87	1.39	**5.32 (1.59; 9.04)**	**0.56 (0.14; 0.98)**	**3.49 (0.07; 6.93)**	0.34 (−0.04; 0.73)
Decentering										
T0	33.02	0.19	33.12	0.18	32.83	0.20				
T1 imputed	38.18	0.74	38.59	0.64	36.28	0.83	1.90 (−0.32; 4.13)	0.35 (−0.08; 0.79)	**2.31 (0.34; 4.28)**	**0.43 (0.04; 0.83)**
T2 imputed	38.84	0.83	38.40	0.72	35.66	0.98	**3.18 (0.64; 5.72)**	**0.52 (0.07; 0.96)**	**2.75 (0.40; 5.09)**	**0.44 (0.03; 0.85)**
Self−compassion										
T0	48.25	0.33	50.71	0.38	46.56	0.39				
T1 imputed	56.72	1.47	56.63	1.35	52.87	1.63	3.84 (−0.56; 8.24)	0.40 (−0.03; 0.84)	3.76 (−0.32; 7.84)	0.47 (0.08; 0.87)
T2 imputed	57.58	1.48	55.33	1.21	52.23	1.57	**5.36 (1.03; 9.68)**	**0.50 (0.07; 0.93)**	3.09 (−0.89; 7.09)	0.47 (0.06; 0.88)
Well−being										
T0	2.88	0.02	2.81	0.02	2.83	0.03				
T1 imputed	3.29	0.09	3.09	0.09	3.03	0.11	0.26 (−0.03; 0.55)	0.34 (−0.08; 0.76)	0.07 (−0.20; 0.34)	0.06 (−0.32; 0.44)
T2 imputed	3.17	0.10	3.08	0.08	3.12	0.10	0.06 (−0.22; 0.33)	0.11 (−0.31; 0.52)	−0.04 (−0.29; 0.23)	0.06 (−0.44; 0.33)

*Note:* Values in bold are significant at the 0.05 level. M and SE are adjusted for the covariates: stratification variables (breast cancer vs. other and curative vs. palliative treatment intent) and baseline of the respective questionnaire.

Abbreviations: CI, confidence interval; M, mean; N, number of subjects; SE, standard error.

*SE for the imputed means.

Differences in HADS total scores between the three groups at the 3 months follow‐up were also significant (*F*‐values for imputed data sets ranging from 0.95 to 9.2), with partial eta squared values ranging from 0.010 to 0.092, and p values ranging from 0.004 to 0.001. Post‐hoc pairwise comparisons showed that group‐blended eMBCT had significantly lower HADS scores than CAU, as did individual‐unguided eMBCT. See Table 3.

In per‐protocol analyses, group effects on HADS scores were significant at both post‐treatment (F (1,113) = 5.01, p = 0.008) and 3 months follow‐up (F (1,112) = 7.99, p < 0.001). Pairwise comparisons showed that group‐blended eMBCT had significantly lower HADS scores than CAU both at post‐treatment and at follow‐up, as did individual‐unguided (Supporting Information S1).

For secondary outcomes, ANCOVA on the ITT sample at post‐treatment (Table 3) showed a significant main effect of group on decentering, with higher post‐treatment scores in individual‐unguided eMBCT compared to CAU. No other significant differences in secondary outcomes were found between groups at post‐treatment.

At 3 months follow‐up, ANCOVA on the ITT sample showed a significant main effect of group on rumination, mindfulness skills, decentering, and self‐compassion. Participants in group‐blended eMBCT reported significantly less rumination and significantly higher mindfulness skills, decentering, and self‐compassion than those in CAU. Participants in individual‐unguided eMBCT also reported significantly less rumination and significantly higher mindfulness skills and decentering than those in CAU (see Table 3).

The per‐protocol analysis revealed a broader range of significant effects compared to the ITT analysis. For more information about secondary outcomes in the per‐protocol sample, see Supplementary Material Results and Table S2.

### 3.5 Mediation Analyses

Mediation analyses in the per‐protocol sample showed no significant results for predicting psychological distress at post‐treatment in either eMCBT condition based on mid‐treatment rumination, mindfulness, decentering, or self‐compassion scores. See Supporting Information S1. However, when using post‐treatment scores for the mediators, significant indirect effects were found for all mediators in both intervention conditions when tested individually. In a model including all mediators, decrease in rumination and improvement in self‐compassion at post‐treatment mediated reductions in psychological distress at 3 months follow‐up for people in the group‐blended eMBCT and only decrease in rumination in the individual‐unguided eMBCT (see Supporting Information S1).

## 4 Discussion

Our trial evaluated the effects of group‐blended and individual‐unguided eMBCT versus CAU on psychological distress in people with cancer and survivors. Post‐treatment, only participants in the group‐blended eMBCT reported significantly lower psychological distress than those receiving CAU. However, at 3 months follow‐up, both eMBCT groups reported significantly lower psychological distress compared to CAU. Per‐protocol analysis (≥ four sessions completed) indicated that participants in the individual‐unguided eMBCT reported less psychological distress and rumination, and greater decentering, self‐compassion, and mindfulness skills at both post‐treatment and follow‐up, compared to CAU. Group‐blended eMBCT participants showed the same benefits, in addition to post‐treatment reduction in fatigue and improvement in well‐being.

The Buddy intervention was designed to address the lack of peer support experienced in a previous therapist‐assisted eMBI for people with cancer [8]. Findings indeed suggest a higher adherence in the group‐blended eMBCT (84%) than in the previous individual therapist‐assisted eMBCT (59%). Interestingly, both eMBCT conditions in our trial also showed greater ES at follow‐up for psychological distress (*d =* 0.64 for group‐blended, *d =* 0.48 for individual unguided) than the face‐to‐face group MBCT in that previous trial (*d =* 0.45 at post‐treatment), indicating more sustained benefits. These results may reflect the co‐creation of the intervention with stakeholders, incorporating personalization, synchronous communication, and engaging design to enhance adherence and outcomes. The group‐blended format also provided teacher and peer support, fostering accountability, shared experience, and emotional validation, factors known to boost engagement and sustain psychological benefits [31].

Face‐to‐face MBIs have shown previously to reduce psychological distress in people with cancer post‐treatment and at follow‐up (Hedges's g = 0.32 and 0.19, respectively) [7]. Significant effects have also been observed at either time point for secondary outcomes such as anxiety, depression, fear of cancer recurrence, fatigue, sleep disturbances, and pain (*g* = 0.20–0.51) [7]. Similarly, recent meta‐analyses have shown that eMBIs improve quality of life, sleep, and well‐being in people with cancer, while reducing anxiety, depression, and stress compared to waitlist and CAU (SMD = −2.21 to −0.30) [32, 33]. Buddy's ES are similar, and for participants who completed ≥ four sessions, we found larger ES than previously reported. Buddy showed strong potential for people with cancer, with greater benefits linked to higher session completion. Enhancing adherence through personalized strategies (e.g., adaptive treatment plans, booster sessions, psychological profiling) could further optimize their impact in cancer care.

Mediation effects were significant only at 3 months follow‐up, suggesting that changes from eMBCT may require time to fully manifest. The BeMind study found that improvements in mindfulness skills, fear of cancer recurrence, and rumination during eMBCT predicted lower psychological distress at 9 months [7]. In this study, rumination and self‐compassion mediated treatment effects in the group‐blended condition, while only rumination did so in the individual‐unguided condition. Thorough mediation analyses in mindfulness research are crucial, including clear identification of mediators, use of multiple models, and appropriate timing [34].

Self‐compassion exhibited the largest ES at post‐treatment, with benefits persisting at follow‐up. In the group‐blended condition, it mediated reductions in psychological distress. A recent meta‐analysis also found that MBIs significantly boost self‐compassion in people with cancer both post‐treatment and at follow‐up [35]. Longitudinal studies show that higher self‐compassion at cancer diagnosis predicts lower depression, anxiety, and fatigue during treatment [36]. These findings underscore the key role of self‐compassion in the well‐being of people with cancer. The lack of studies focusing on compassion‐based interventions for this population highlights an opportunity for future research.

### 4.1 Clinical Implications

Guided digital psychotherapy is generally more effective than unguided interventions [48]. A meta‐analysis on online cognitive behavioral therapies for depression found greater immediate benefits with guided therapy than unguided therapy, though differences diminished over time (the guided showed a strong initial effect that decreased over time, while the unguided started with a smaller effect but remained stable or slightly improved) [37]. Similarly, our study showed that group‐blended eMBCT reduced psychological distress at post‐treatment, whereas both interventions demonstrated benefits at follow‐up. This suggests that guidance may provide immediate psychological relief, while unguided interventions may result in more gradual, delayed effects.

Participants in individual‐unguided eMBCT completed fewer sessions and had lower engagement than those in group‐blended eMBCT. In our nonrandomized feasibility study, all 12 initial participants opted for the group‐blended eMBCT [10]. Structured guidance may be an important facilitator for sustaining participation and engagement. In fact, it has been shown that counselor interaction and dialogue support significantly improve adherence in web‐based interventions [31].

Although adherence to individual‐unguided eMBCT was lower than group‐blended eMBCT, it exceeded the typical 50% adherence rate for eHealth interventions [31]. Higher adherence is common in interventions for chronic conditions and in those incorporating persuasive technology or other engaging design features [31]. During the co‐creation process, we integrated stakeholder feedback, including two avatar choices, film clips of past participants, automatic feedback, and various meditation voices. We also pilot‐tested the intervention before the full‐scale RCT. The co‐creation and pilot testing aimed at identifying barriers and addressing limitations, likely contributing to the trial's above‐average adherence.

The difference in timing of the effect between the intervention conditions may have practical implications. Group‐blended eMBCT may be more suitable for people with cancer requiring immediate psychological relief, such as people with advanced cancer, who often experience higher levels of distress and might benefit from the support of a mindfulness teacher. In contrast, the individual‐unguided format offers flexibility for those preferring a self‐paced approach. While both eMBCT formats have potential for integration into the healthcare system, the individual‐unguided eMBCT provides a more scalable and resource‐efficient option. A sustainable and effective implementation of both eMBCTs in oncological care requires pilot testing in diverse clinical settings and standardized training for healthcare providers for the group‐blended condition.

### 4.2 Study Strengths and Limitations

Strengths of our trial include building on the foundations of our previous study, which has been recognized as a robust contribution to psycho‐oncology in recent years [38]. The content and format of the eMBCT was thoroughly developed through a co‐creation process involving people with cancer and experts in mindfulness, oncology, and eHealth. We also incorporated improvements from a prior feasibility study [11] and the intervention was delivered by experienced mindfulness teachers under supervision.

This study also has several limitations. Although we strived for inclusivity, the sample was predominantly composed of highly educated white women, a common trend in mindfulness intervention research [39], limiting generalizability. Plus, people with a type of cancer other than breast and under palliative care dropped out more. Furthermore, the original target sample size was not achieved, leaving some uncertainty about the interventions' effectiveness. Additionally, we did not collect explicit information on participants' current treatment status. Although some treatment‐related details were obtained through open‐ended questions, they were insufficient for meaningful analysis. Finally, the study was not powered to directly compare effects of group‐blended and individual‐unguided eMBCT.

## 5 Conclusion

Group‐blended and individual‐unguided eMBCT can benefit people with cancer and survivors. Both formats offer flexibility in terms of time, location, and travel, and require fewer resources than face‐to‐face MBIs. Future research will explore the long‐term effects, moderators, and cost‐effectiveness of both eMBCT formats to strengthen evidence and support the integration of eMBIs into oncological care.

## Author Contributions

**Nasim Badaghi:** conceptualization, data curation, formal analysis, investigation, validation, visualization, writing (draft, review, and editing). **Linda Kwakkenbos:** conceptualization, investigation, project administration, supervision, validation, writing (review and editing). **Judith Prins:** funding acquisition, methodology, supervision, writing (review and editing). **Rogier Donders:** formal analysis, validation. JP: conceptualization, funding acquisition, methodology, supervision, writing (review and editing). **Saskia Kelders:** conceptualization, funding acquisition, methodology, supervision, writing (review and editing). **Anne Speckens:** conceptualization, funding acquisition, methodology, project administration, supervision, writing (review and editing).

## Conflicts of Interest

The authors declare no conflicts of interest.

## Supporting information


Supporting Information S1
