# Supplementary material for: Randomized Controlled Trial of Group‐Blended and Individual‐Unguided Online Mindfulness‐Based Cognitive Therapy to Reduce Psychological Distress in People With Cancer
Source: Psychooncology. 2025 Sep 19;34(9):e70286. doi: 10.1002/pon.70286 (PMC12447259; doi:10.1002/pon.70286)
Supplement: Supplementary file 1 — Supporting Information S1 [file PON-34-e70286-s001.docx]

# Supplementary Material

Contents

Statistical analysis plan (SAP) 2

Amendments to the trial protocol 7

Supplementary Table S1**.** Mean item and total scores for TWEETS for group-blended and individual-unguided eMBCT.8

Results from the per-protocol sample (participants who completed 4 sessions or more) 9

Supplementary Table S2. Trial outcomes at baseline, post-intervention, and 3-month follow-up (per-protocol; 4 sessions or more) 11

Supplementary Table S3. Mediation results of individual mediation models per mediator per group (post-treatment)13

Supplementary Table S4. Mediation results of individual mediation models per mediator per group (follow-up)14

Supplementary Table S5. Mediation results of mediation models with all mediators combined per group (follow-up)15

**Statistical Analysis Plan**

Statistical Analysis Plan (SAP) for the *Randomized controlled trial of group-blended and individual-unguided online mindfulness-based cognitive therapy to reduce psychological distress in people with cancer*.

**Administrative Information**

Registration: Dutch Registry CCMO, NL73117.091.20; clinicaltrials.gov, NCT05336916

Protocol: https://bmcpsychology.biomedcentral.com/articles/10.1186/s40359-023-01052-2

Pilot Study: https://formative.jmir.org/2024/1/e52338

SAP Version 1 (September 27^th^, 2024)

This SAP was drafted by Nasim Badaghi and Linda Kwakkenbos. Anne Speckens, Judith Prins, and Saskia Kelders revised and agreed with the final version. Rogier Donders is the senior statistician who provided guidance to the researchers for all analyses.

**SAP Version 1**

*Buddy (effectiveness of group-blended and individual-unguided delivery of mindfulness-based cognitive therapy (MBCT) versus care as usual (CAU)) is an intervention aimed at reducing psychological distress in people with cancer and cancer survivors. Buddy consists of two conditions of online MBCT (eMBCT): group-blended and individual-unguided. Both conditions have identical content and duration but different format.*

*The Buddy Trial is a three-arm, parallel, single-center randomized controlled trial (RCT). It is a blocked RCT because eligible participants where first stratified based on type of cancer (breast versus other) and treatment intention (palliative versus curative), and then randomized into group-blended, individual-unguided, or CAU using blocks of 6, 12, 18 (varying).*

*The primary outcome analysis compares psychological distress, measured by the total scores on the Hospital Anxiety and Depression Scale (HADS), between participants randomly assigned to the group-blended or individual-unguided conditions with CAU, after intervention completion (approximately three months post-baseline). Secondary outcomes will include HADS post-treatment, as well as the following outcome measures post-treatment and at three months follow-up:*

- Fear of Cancer Recurrence Inventory – severity subscale (FCRI-SF): fear of cancer recurrence.
- Checklist Individual Strength – fatigue severity subscale (CIS): fatigue.
- Rumination and Reflection Questionnaire – rumination subscale (RRQ): rumination.
- Five Facet Mindfulness Questionnaire Short Form (FFMQ-SF): mindfulness skills.
- Experiences Questionnaire – decentering subscale (EQ): decentering.
- Self-Compassion Scale Short Form (SCS-SF): self-compassion.
- Mental Health Continuum-Short Form (MHC-SF): positive mental health.
- Twente Engagement with Ehealth Technologies Scale (TWEETS): engagement with the intervention.

RRQ, FFMQ-SF, EQ, and SCS-SF will be also assessed at mid-treatment (four weeks after starting the intervention). Detailed information about each outcome measure can be found in the previously published protocol paper^[[1]](#footnote-1)^.

**Statistical Analyses**

- Data will be checked for completeness and cleaned

- Descriptive Analyses:

- For the total sample and by arm (group-blended, individual-unguided, and CAU), we will describe demographic and disease characteristics (see draft Table 1).

*Primary Analysis*

All analyses will be conducted in SPSS and will be two-sided, using an alpha value of 0.05.

The primary outcome analysis will compare psychological distress at post-treatment (T1), assessed with the total scores from HADS between participants in the group-blended and CAU arms and between the individual-unguided and CAU arms at post-treatment (T1). We will use a closed testing procedure with ANCOVA. Group (group-blended eMBCT, individual-unguided eMBCT, and CAU) will be added as between subject factor, and baseline HADS scores and stratification variables (type of cancer and treatment intent) will be added as covariates.

After, we will conduct pairwise comparisons between group-blended eMBCT versus CAU, and individual-unguided eMBCT versus CAU.

All statistical analyses will use an intention to treat (ITT) approach to account for missing data. We will use multiple imputation by chained equations to generate 20 imputed datasets, using 15 cycles per imputed dataset using the mice package^[[2]](#footnote-2)^, with minimum and maximum boundaries for the imputed values based on the questionnaire score range (e.g., HADS: 0-42). Variables in the mice procedure will include:

- Trial variables: intervention arm (group-blended, individual-unguided, and CAU).
- Stratification variables: type of cancer (breast versus other), treatment intent (palliative versus curative).
- Measures of all primary and secondary outcomes at baseline, mid-treatment*,* post-treatment, and three months follow-up.
- Auxiliary variables: mid-treatment questionnaires (rumination (RRQ), mindfulness skills (FFMQ-SF), decentering (EQ), and self-compassion (SCS-SF).

*Secondary Analyses*

Analyses of secondary outcomes at post-treatment (T1) will be conducted using the same procedure as in the primary analysis but with the secondary outcome measures. In addition, we will conduct the same analyses (ANCOVA with pairwise comparisons) at three months follow-up.

*Mediation Analysis*

We will use the per-protocol sample (participants who completed four or more sessions) for mediation analyses. Mid-treatment assessments will be used to examine whether changes in potential mediators —rumination (RRQ), mindfulness (FFMQ), decentering (EQ), self-compassion (SCS-SF) — during the first half of the training predict changes in psychological distress (HADS) over the full duration of the training. We will follow the recommendations of Preacher and Hayes^[[3]](#footnote-3)^ for multiple mediation models. Each potential mediator will first be tested individually, followed by a model that includes all mediators together. Separate mediation models will be conducted for each comparison: group-blended versus CAU and individual-unguided versus CAU.

**Draft Table 1. Baseline sociodemographic and clinical characteristics.**

| Characteristic | All  (n = 186) | Group, blended  (n = 57) | Individual, unguided  (n = 75) | CAU  (n = 54) |
| --- | --- | --- | --- | --- |
| Sex, N (%)  Female  Male |  |  |  |  |
| Age, years (SD)  Mean |  |  |  |  |
| Diagnosis, N (%)  Breast cancer  Blood cancer  Skin cancer  Intestine cancer  Colon cancer  Ovarian cancer  Prostate cancer  Other |  |  |  |  |
| Anticancer treatment, N (%)  Curative  Palliative |  |  |  |  |
| Occupation, N (%)  Employed  Retired  On disability  Houseman/-wife  Unemployed  Other (not specified) |  |  |  |  |
| Level of Education, N (%)  High  Middle  Low  Other (not specified) |  |  |  |  |
| Time since diagnosis, N (%)  1-3  3-6  >6 |  |  |  |  |
| Marital Status, N (%)  Married or partnership  Unmarried  Divorced  Widow |  |  |  |  |

**Amendments to SAP (March, 2024)**

The following was added to the statistical analyses:

Outcome analyses used ANCOVA to compare intervention effects at post-treatment and follow-up, adjusting for baseline and stratification variables. ANCOVA was chosen for its direct and interpretable estimates at these fixed timepoints, aligning with the trial’s focus.

*Primary Analyses*

We will run ANCOVAs on a second imputed data set generated through predictive mean matching (PMM). This approach will enable us to compare the results from two models and assess the accuracy and consistency of the imputed data.

*Mediation Analyses*

For mediation analyses at post-treatment we will use psychological distress (HADS) at post-treatment as the dependent variable, the assessment at mid-treatment for the potential mediators (including rumination (RRQ), mindfulness (FFMQ), decentering (EQ), self-compassion (SCS-SF)), and the intervention condition as independent variable. Baseline of the outcome and mediators will be used as covariates to model difference scores.

For mediation analyses at follow-up we will use we will use psychological distress (HADS) at follow-up as the dependent variable, the assessment at post-treatment for the potential mediators (including rumination (RRQ), mindfulness (FFMQ), decentering (EQ), self-compassion (SCS-SF)), and the intervention condition as independent variable. Baseline of the outcome and mediators will be used as covariates to model difference scores.

**Amendments to the trial protocol**

*Randomization (as published in the protocol)^1^*

Upon the start of the trial, participants were randomized to blended versus unguided versus CAU with a 1:2:1 ratio to one of the three groups because of anticipated higher drop-out rate in the unguided eMBCT. However, upon inspection of completion rates after one year of enrollment, it appeared that the drop-out rates were quite similar across the three conditions. Therefore, the remainder of participants are randomly assigned with a 1:1:1 ratio.

**Supplementary Table S1.** Mean item for TWEETS for group-blended and individual-unguided eMBCT.

| **Current Engagement (mid-treatment)** | Group-blended (n = 46) | Individual-unguided (n = 57) |
| --- | --- | --- |
|  | mean (SD) | mean (SD) |
| 1. Using this training has become part of my daily routine. | 2.22 (0.99) | 2.02 (1.04) |
| 1. This training is easy to use. | 2.41 (0.86) | 2.28 (0.92) |
| 1. I use this training as often as necessary (to achieve my goals). | 2.48 (0.89) | 2.39 (0.94) |
| 1. The training makes it easier for me to work on my goals. | 2.67 (0.76) | 2.61 (0.94) |
| 1. This training motivates me to achieve my goals. | 2.65 (0.82) | 2.60 (0.92) |
| 1. This training helps to gain more insight. | 3.15 (0.89) | 2.93 (0.90) |
| 1. I enjoy using this training. | 2.72 (0.86) | 2.49 (0.97) |
| 1. It is nice to see my progress in this training. | 2.80 (0.81) | 2.60 (0.79) |
| 1. This training suits me as a person. | 2.37 (1.02) | 2.51 (1.02) |
|  |  |  |
| **Past Engagement (post-treatment, T1)** | Group-blended (n = 41) | Individual-unguided (n = 51) |
|  | mean (SD) | mean (SD) |
| 1. Using this training became part of my daily routine. | 2.44 (1.14) | 2.14 (1.09) |
| 1. This training was easy to use. | 2.71 (0.84) | 2.41 (1.00) |
| 1. I used this training as often as necessary (to achieve my goals). | 2.56 (1.03) | 2.41 (0.98) |
| 1. This training made it easier for me to work on my goals. | 2.88 (0.75) | 2.65 (0.82) |
| 1. This training motivated me to achieve my goals. | 2.95 (0.74) | 2.57 (0.99) |
| 1. This training helped to gain more insight. | 3.24 (0.62) | 2.96 (0.75) |
| 1. I enjoyed using this training. | 2.98 (0.99) | 2.69 (0.84) |
| 1. I enjoyed seeing the progress I made in this training. | 3.00 (0.67) | 2.55 (0.83) |
| 1. This training suits me as a person. | 2.59 (1.18) | 2.39 (1.12) |

**Results from the per-protocol sample**

An ANCOVA was conducted to assess differences between the intervention arms at post-treatment and three months follow-up. Pairwise comparisons were used to determine whether the differences between group-blended eMBCT and CAU, as well as individual-unguided eMBCT and CAU, were statistically significant across each outcome measure per time point. For the number of participants in the per-protocol sample who completed each questionnaire per time point, see Supplementary Table S2.

At post-treatment, results from the ANCOVA on the per-protocol sample (four sessions or more) showed a significant main effect of intervention group on psychological distress, fatigue, rumination, mindfulness skills, decentering, self-compassion, and well-being (Supplementary Table S2). Results from the pairwise comparisons showed that at post-treatment, participants in the group-blended eMBCT had a significantly larger decrease in psychological distress at post-treatment (ES=0.64), as well as a greater reduction of fatigue (ES=0.39) and rumination (ES= 0.33), and increase in decentering (ES=0.62), self-compassion (ES=0.66) and well-being (ES=0.62) when compared to CAU. Participants in the individual-unguided eMBCT had a significantly larger decrease in psychological distress (ES=0.56) and rumination (ES=0.61), and a significantly greater increase in mindfulness skills (ES=0.36), decentering (ES=0.61), and self-compassion (ES=0.83) at post-treatment when compared to CAU.

At follow-up, results from the ANCOVA on the per-protocol sample showed a significant main effect of intervention group on psychological distress, rumination, mindfulness skills, decentering, and self-compassion. Results from the pairwise comparisons showed that at follow-up, participants in the group-blended eMBCT had a significantly larger decrease in psychological distress (ES=0.71) and rumination (ES=0.43), and a greater increase in mindfulness skills (ES=0.71), decentering (ES=0.55), and self-compassion (ES=0.53) when compared to CAU. Participants in the individual-unguided eMBCT had a significant larger decrease in psychological distress (ES=0.56) and rumination (ES=0.61), and a significant greater increase in mindfulness skills (ES=0.47), decentering (ES=0.56), and self-compassion (ES=0.63) at follow-up when compared to CAU. See Supplementary Table S2.

**Supplementary Table S2.** Primary and secondary outcome measures at baseline, post-treatment, and three months follow-up (per-protocol; four sessions or more).

|  |  | Group, blended eMBCT | |  |  | GROUPS  Individual, unguided eMBCT |  |  |  |  |  |  | CAU-Group, blended eMBCT |  | | |
| --- | --- | --- | --- | --- | --- | --- | --- | --- | --- | --- | --- | --- | --- | --- | --- | --- |
|  |  | |  |  |  |  |  |  | CAU |  |  |  | | | PAIRWISE COMPARISONS |  |
| Outcome measure | N | | M | SD | N | M | SD | N | M | SD | Mean difference (95% CI) | ES (95% CI) | | | Mean difference | CAU-Individual, unguided eMBCT  ES (95% CI) |
| Psychological distress  T0  T1 original  T2 original | 57  41  42 | | 15.35  10.44  9.57 | 6.62  5.01  5.22 | 75  42  41 | 15.51  11.57  10.61 | 6.85  7.04  4.76 | 54  36  35 | 16.31  14.47  13.80 | 6.51  7.52  6.67 | **- 3.55 (- 5.86; - 1.25)**  **- 3.66 (- 5.61; - 1.71)** | **0.64 (0.18; 1.09)**  **0.71 (0.25; 1.18)** | | | **- 2.69 (- 4.98; - 0.40)**  **- 3.19 (- 5.15; - 1.24)** | 0.39 (- 0.05; 0.85)  **0.56 (0.09; 1.02)** |
| Fear of cancer recurrence  T0  T1 original  T2 original | 57  40  42 | | 80.14  69.58  66.88 | 22.49  19.89  19.67 | 75  41  40 | 77.58  72.24  73.70 | 24.85  22.13  23.46 | 54  35  34 | 77.28  72.50  71.94 | 22.72  20.05  20.09 | - 3.07 (- 8.82; 2.68)  - 4.64 (- 11.09; 1.82) | 0.13 (- 0.32; 0.59)  0.16 (- 0.30; 0.61) | | | - 0.11 (- 5.82; 5.60)  - 0.50 (- 7.04; 6.04) | 0.00 (- 0.45; 0.45)  - 0.17 (- 0.63; 0.29) |
| Fatigue severity  T0  T1 original  T2 original | 57  41  42 | | 38.05  31.76  30.36 | 11.72  12.04  12.31 | 75  41  40 | 35.72  32.85  32.07 | 11.86  11.95  11.19 | 54  36  35 | 38.31  36.42  34.17 | 10.64  11.56  11.24 | **- 3.87 (- 7.62; - 0.11)**  - 2.70 (- 6.78; 1.38) | 0.39 (- 0.06; - 0.85)  0.32 (- 0.13; 0.77) | | | - 1.45 (- 2.33; 5.22)  - 0.88 (- 5.01; 3.24) | 0.30 (- 0.15; 0.75)  0.19 (- 0.27; 0.64) |
| Rumination  T0  T1 original  T2 original | 57  40  42 | | 3.40  3.06  3.07 | 0.68  0.74  0.77 | 75  41  40 | 3.28  2.95  2.93 | 0.86  0.74  0.78 | 54  34  33 | 3.43  3.29  3.38 | 0.62  0.64  0.70 | **- 0.24 (- 0.46; - 0.03)**  **- 0.40 (- 0.66; - 0.15)** | 0.33 (- 0.13; 0.79)  0.43 (- 0.03; 0.89) | | | **- 0. 23 (- 0.44; - 0.01)**  **- 0.45 (- 0.69; - 0.19)** | **0.49 (0.03; 0.95)**  **0.61 (0.13; 1.07)** |
| Mindfulness skills  T0  T1 original  T2 original | 57  40  42 | | 79.53  85.40  87.71 | 10.77  10.29  10.24 | 75  41  40 | 78.15  85.46  84.93 | 11.18  11.12  10.21 | 54  34  34 | 77.77  81.09  79.56 | 12.38  11.67  12.79 | 2.87 (- 0.76; 6.50)  **7.61 (3.89; 11.32)** | 0.39 (- 0.07; 0.85)  **0.71 (0.24; 1.18)** | | | **4.25 (0.66; 7.85)**  **5.98 (2.22; 9.73)** | 0.39 (- 0.08; 0.84)  **0.47 (0.01; 0.93)** |
| Decentering  T0  T1 original  T2 original | 57  40  42 | | 33.02  38.93  38.83 | 6.57  5.48  5.88 | 75  41  40 | 33.12  39.07  39.98 | 7.02  5.96  6.49 | 54  34  33 | 32.83  35.50  35.27 | 6.76  5.67  7.26 | **3.59 (1.35; 5.83)**  **4.37 (1.91; 6.83)** | **0.62 (0.15; 1.08)**  **0.55 (0.08; 1.01)** | | | **3.49 (1.37; 5.82)**  **4.23 (1.75; 6.69)** | **0.61 (0.15; 1.08)**  **0.56 (0.10; 1.02)** |
| Self-compassion  T0  T1 original  T2 original | 57  40  42 | | 48.25  57.70  57.81 | 11.40  11.01  13.54 | 75  41  40 | 50.71  60.07  58.73 | 15.35  12.40  12.09 | 54  34  33 | 46.56  49.79  50.73 | 13.15  12.86  13.20 | **6.64 (2.53; 10.75)**  **7.39 (3.15; 11.64)** | **0.66 (0.19; 1.13)**  **0.53 (0.06; 0.99)** | | | **6.17 (2.02; 10.33)**  **5.33 (1.02; 9.64)** | **0.82 (0.34; 1.28)**  **0.63 (0.16; 1.11)** |
| Well-being  T0  T1 original  T2 original | 57  40  42 | | 2.88  3.45  3.30 | 0.87  0.72  0.87 | 75  41  40 | 2.81  3.05  3.04 | 0.96  0.93  0.97 | 54  35  34 | 2.83  2.92  3.08 | 0.94  0.99  0.69 | **0.35 (0.05; 0.64)**  0.17 (- 0.12; 0.45) | **0.62 (0.15; 1.08)**  0.26 (- 0.19; 0.72) | | | 0.18 (- 0.11; 0.46)  0.12 (- 0.16; 0.41) | 0.14 (- 0.31; 0.59)  0.06 (- 0.52; 0.40) |

Abbreviations: N, number of subjects M, mean; SE, standard error; CI, confidence interval.

Values in bold are significant at the 0.05 level.

*M and SE are adjusted for the covariates: stratification variables (breast cancer versus other and curative versus palliative treatment intent) and baseline of the respective questionnaire.

**Supplementary Table S3.** Mediation results of individual mediation models per mediator per group (post-treatment).

| Group | Mediator | Indirect Effect of  the mediator | SE | 95% CI |
| --- | --- | --- | --- | --- |
| Group-blended versus CAU | RRQ | - 0.32 | 0.43 | - 1.37; 0.29 |
|  | FFMQ-SF | 0.21 | 0.39 | - 0.69; 0.96 |
|  | EQ | - 0.01 | 0.27 | - 0.53; 0.66 |
|  | SCS-SF | 0.09 | 0.20 | - 0.56; 0.31 |
| Individual-unguided versus CAU | RRQ | - 0.04 | 0.17 | - 0.33; 0.39 |
|  | FFMQ-SF | 0.07 | 0.12 | - 0.16; 0.34 |
|  | EQ | 0.00 | 0.14 | - 0.29; 0.31 |
|  | SCS-SF | 0.00 | 0.07 | - 0.18; 0.15 |

Abbreviations: SE, standard error; CI, confidence interval; HADS, Hospital Anxiety and Depression scale; RRQ, Rumination and Reflection Questionnaire

(rumination subscale); FFMQ-SF, Five Facet Mindfulness Questionnaire Short Form; EEQ, Experiences Questionnaire (decentering subscale);

SCS-SF, Self-Compassion Scale Short Form.

Mid-treatment assessments (after week four) were used to examine whether changes in potential mediators —rumination (RRQ), mindfulness (FFMQ), decentering (EQ), self-compassion (SCS-SF) — during the first half of the training predicted changes in psychological distress (HADS) over the full duration of the training (post-treatment). Supplementary Table S2 shows the results of the mediation model when each mediator was individually tested. None of the indirect effects of the tested mediators statistically significant. This was the case for both the group-blended eMBCT versus CAU and the individual-unguided eMBCT versus CAU comparisons.

**Supplementary Table S4.** Mediation results of individual mediation models per mediator per group (follow-up).

| Group | Mediator | Indirect Effect of  the mediator | SE | 95% CI |
| --- | --- | --- | --- | --- |
| Group-blended versus CAU | RRQ | - 1.55 | 0.49 | - 2.58; - 0.62 |
|  | FFMQ-SF | - 0.98 | 0.50 | - 2.07; - 0.13 |
|  | EQ | - 1.21 | 0.64 | - 2.71; - 0.14 |
|  | SCS-SF | - 1.53 | 0.64 | - 2.98; - 0.49 |
| Individual-unguided versus CAU | RRQ | - 0.71 | 0.24 | - 1.20; - 0.21 |
|  | FFMQ-SF | - 0.65 | 0.27 | - 1.22; - 0.17 |
|  | EQ | - 0.79 | 0.31 | - 1.46; - 0.25 |
|  | SCS-SF | - 0.84 | 0.33 | - 1.59; - 0.28 |

Abbreviations: SE, standard error; CI, confidence interval; HADS, Hospital Anxiety and Depression scale; RRQ, Rumination and Reflection Questionnaire

(rumination subscale); FFMQ-SF, Five Facet Mindfulness Questionnaire Short Form; EEQ, Experiences Questionnaire (decentering subscale);

SCS-SF, Self-Compassion Scale Short Form.

Post-treatment assessments were used to examine whether changes in potential mediators —rumination (RRQ), mindfulness (FFMQ), decentering (EQ), self-compassion (SCS-SF) — after the eMBCT training predicted changes in psychological distress (HADS) at three months follow-up. Supplementary Table S3 shows the results of the mediation model when each mediator was individually tested. All tested mediators showed significant indirect effects in both the group-blended eMBCT versus CAU and the individual-unguided eMBCT versus CAU comparisons. The strongest indirect effects were observed for self-compassion (SCS-SF) and rumination (RRQ) in the group-blended eMBCT condition, while all mediators also demonstrated significant effects in the individual-unguided eMBCT condition, though with smaller effect sizes.

**Supplementary Table S5.** Mediation results of mediation models with all mediators combined per group (follow-up).

| Group | Mediator | Indirect Effect of  the mediator | SE | 95% CI |
| --- | --- | --- | --- | --- |
| Group-blended versus CAU | RRQ | - 0.75 | 0.54 | - 2.08; - 0.05 |
|  | FFMQ-SF | - 0.56 | 0.40 | - 1.41; 0.19 |
|  | EQ | 0.12 | 0.69 | - 1.19; 1.68 |
|  | SCS-SF | - 1.04 | 0.64 | - 2.50; - 0.01 |
| Individual-unguided versus CAU | RRQ | - 0.34 | 0.19 | - 0.88; - 0.02 |
|  | FFMQ-SF | - 0.20 | 0.16 | - 0.56; 0.07 |
|  | EQ | - 0.29 | 0.25 | - 0.84; 0.15 |
|  | SCS-SF | - 0.35 | 0.25 | - 0.92; 0.03 |

Abbreviations: SE, standard error; CI, confidence interval; HADS, Hospital Anxiety and Depression scale; RRQ, Rumination and Reflection Questionnaire

(rumination subscale); FFMQ-SF, Five Facet Mindfulness Questionnaire Short Form; EEQ, Experiences Questionnaire (decentering subscale);

SCS-SF, Self-Compassion Scale Short Form.

Post-treatment assessments were used to examine whether changes in potential mediators —rumination (RRQ), mindfulness (FFMQ), decentering (EQ), self-compassion (SCS-SF) — after the eMBCT training predicted changes in psychological distress (HADS) at three months follow-up. Supplementary Table S4 shows the results of the mediation model with all mediators combined in one model per intervention condition. When including all mediators in a single model, the indirect effects of rumination (RRQ) and self-compassion (SCS-SF) remained significant in both the group-blended and individual-unguided eMBCT conditions, though the effect sizes were generally smaller compared to when mediators were tested separately. In contrast, the indirect effects of mindfulness skills (FFMQ-SF) and decentering (EQ) were no longer significant.

.

1. Badaghi N, van Kruijsbergen M, Prins J, Kelders S, Cillessen L, Compen F, et al. Effect of blended and unguided online delivery of mindfulness-based cognitive therapy versus care as usual on distress among cancer patients and survivors: protocol for the three-arm parallel randomized controlled buddy trial. *BMC Psychology*. 2023;11(1). doi.org/10.1186/s40359-023-01052-2. [↑](#footnote-ref-1)
2. van Buuren S, Groothuis-Oudshoorn K. mice: Multivariate Imputation by Chained Equations in R. J Stat Softw. 2011; 45:1-67 Preacher KJ, Hayes AF. Asymptotic and resampling strategies for assessing and comparing indirect effects in multiple mediator models. Behav Res Methods. 2008; 40(3):879–91. [↑](#footnote-ref-2)
3. Preacher KJ, Hayes AF. Asymptotic and resampling strategies for assessing and comparing indirect effects in multiple mediator models. Behav Res Methods. 2008; 40(3):879–91. [↑](#footnote-ref-3)
